# EZH2 Codon 641 Mutations are Common in BCL2-Rearranged Germinal Center B Cell Lymphomas

**DOI:** 10.1371/journal.pone.0028585

**Published:** 2011-12-14

**Authors:** Russell J. H. Ryan, Mai Nitta, Darrell Borger, Lawrence R. Zukerberg, Judith A. Ferry, Nancy Lee Harris, A. John Iafrate, Bradley E. Bernstein, Aliyah R. Sohani, Long Phi Le

**Affiliations:** 1 Department of Pathology, Massachusetts General Hospital, Boston, Massachusetts, United States of America; 2 Broad Institute of Massachusetts Institute of Technology and Harvard University, Cambridge, Massachusetts, United States of America; 3 Howard Hughes Medical Institute, Chevy Chase, Maryland, United States of America; University of Nebraska – Lincoln, United States of America

## Abstract

Mutations at codon 641 of *EZH2* are recurrent in germinal center B cell lymphomas, and the most common variants lead to altered EZH2 enzymatic activity and enhanced tri-methylation of histone H3 at lysine 27, a repressive chromatin modification. As an initial step toward screening patients for cancer genotype-directed therapy, we developed a screening assay for *EZH2* codon 641 mutations amenable for testing formalin-fixed clinical specimens, based on the sensitive SNaPshot single nucleotide extension technology. We detected *EZH2* mutations in 12/55 (22%) follicular lymphomas (FL), 5/35 (14%) diffuse large B cell lymphomas with a germinal center immunophenotype (GCB-DLBCL), and 2/11 (18%) high grade B cell lymphomas with concurrent rearrangements of *BCL2* and *MYC*. No *EZH2* mutations were detected in cases of Burkitt lymphoma (0/23). *EZH2* mutations were frequently associated with the presence of *BCL2* rearrangement (BCL2-R) in both the FL (28% of BCL-R cases versus 0% of BCL2-WT cases, p<0.05) and GCB-DLBCL groups (33% of BCL2-R cases versus 4% of BCL2-WT cases, p<0.04), and across all lymphoma types excluding BL (27% of BCL2-R cases versus 3% of BCL2-WT cases, p<0.003). We confirmed gain-of-function activity for all previously reported *EZH2* codon 641 mutation variants. Our findings suggest that *EZH2* mutations constitute an additional genetic “hit” in many *BCL2*-rearranged germinal center B cell lymphomas. Our work may be helpful in the selection of lymphoma patients for future trials of pharmacologic agents targeting EZH2 and EZH2-regulated pathways.

## Introduction

Human mature B cell lymphomas encompass a diverse spectrum of biologically and clinically distinct entities, many of which appear to recapitulate a specific stage of B cell development by morphologic, immunophenotypic and/or gene expression criteria. Follicular lymphoma (FL), Burkitt lymphoma (BL), and a gene expression-defined subset of diffuse large B cell lymphoma (germinal center B cell-phenotype diffuse large B cell lymphoma, or GCB-DLBCL) all recapitulate biological features of normal germinal center B cells. [1], [2] As new discoveries have improved our understanding of the genetics and phenotypic characteristics of B cell lymphomas, it has become clear that some oncogenic mutations occur with increased frequency, or even exclusively, in lymphomas of a certain phenotype. One prominent example is the t(14;18)(q32;q21) *IGH-BCL2* rearrangement, which leads to overexpression of the Bcl-2 oncoprotein, and is frequently present in follicular lymphoma and GCB-DLBCL, but not in other gene expression-defined subtypes of DLBCL. [3]

*EZH2* encodes the enzymatic subunit of the polycomb repressive complex 2 (PRC2), which mediates gene repression through trimethylation of histone H3 at lysine 27 (H3K27). Point mutations affecting codon 641 of *EZH2* were recently described in a subset of GCB-DLBCL and FL, [4] but were absent from activated B cell-phenotype DLBCL (ABC-DLBCL), small lymphocytic lymphoma, mantle cell lymphoma, and peripheral T cell lymphoma, all of which lack a germinal center phenotype. Lymphoma-associated mutations affecting *EZH2* were initially reported to result in enzymatic loss-of-function based on *in vitro* studies which used unmodified histone tails as a substrate for modification by recombinant PRC2 complexes, but the most common variants have subsequently been shown to possess increased activity in trimethylation of H3K27 in the mono- or di-methylated state. [5], [6] Overexpression and hyperactivity of *EZH2* have been implicated in the pathogenesis of several cancer types, including prostate, [7], [8] breast, and endometrial carcinomas, as well as melanoma. [9] The discovery that *EZH2* is a mutant oncogene in germinal center-phenotype B cell lymphomas raises the possibility of targeting this pathway pharmacologically in the treatment of patients with these lymphomas. Important steps toward this goal include further delineation of the lymphoma types in which this mutation occurs, and development of assays amenable to detection of these mutations in routine clinical specimens.

In this study, we report the results of testing for *EZH2* codon 641 mutations in a large series of B cell lymphomas of germinal center origin, using a sensitive and specific single nucleotide extension method (SNaPshot) which has been validated for clinical tumor genotyping at our institution. We detect *EZH2* mutations in a significant proportion of *BCL2*-rearranged follicular lymphomas, *BCL2*-rearranged diffuse large B cell lymphomas, and high-grade B cell lymphomas with concurrent *BCL2* and *MYC* rearrangements (so-called “double-hit lymphomas”), but not in BL. The data suggest that this mutation is not universally present in all germinal center-phenotype B cell lymphoma subtypes but appears to correlate with *BCL2* status. Finally, we provide evidence that all previously reported *EZH2* codon 641 mutation variants show the potential for enhanced H3K27 trimethylation activity.

## Materials and Methods

### Ethics statement

All research was conducted with the written approval of the Partners Healthcare Human Research Committee in accordance with the Ethical Principles and Guidelines for the Protection of Human Subjects of Research, generally known as the “Belmont Report.”

### Case selection

Cases of FL, GCB-DLBCL, *MYC* and *BCL2* rearranged “double hit” lymphomas (DHL), and BL were identified from our pathology archives. All cases were diagnosed according to WHO 2008 criteria, and cases of uncertain classification were reviewed by a panel of expert hematopathologists (A.R.S., L.R.Z., J.A.F., and N.L.H.). The follicular lymphoma cohort was enriched for cases of low morphologic grade and a Ki-67 proliferation index (PI) >30%, which prior work has suggested may represent a distinct poor prognosis subgroup. [10] GCB-DLBCL cases were identified on the basis of tumor expression of either CD10 or Bcl-6 without Mum-1. [11] DHL cases were classified as DLBCL or intermediate between DLBCL and BL by WHO 2008 criteria, but were analyzed as one unique group for this study.

### Immunohistochemistry

Immunohistochemistry, including stains for CD10, Bcl-6, Mum-1, and Ki-67, was performed using protocols and antibodies validated for clinical use as described previously. [12]

### DNA extraction

Areas showing at least 40% malignant cells on hematoxylin and eosin (H&E) or immunohistochemically stained slides were selected for coring (1.5 mm) of the respective formalin-fixed, paraffin-embedded (FFPE) tissue blocks. Total nucleic acid (TNA) was purified using the FormaPure method (Agencourt, Danvers, MA), which was automated on a Beckman Biomek II liquid handler (Beckman Coulter, Brea, CA).

### SNaPshot genotyping

Our institution routinely uses the SNaPshot multiplex single-nucleotide extension assay for clinical genotyping of tumor-associated point mutations. [13] We designed polymerase chain reaction (PCR) and SNaPshot extension primers to redundantly genotype the first two nucleotides of *EZH2* codon 641 in both the forward and reverse direction, a strategy that can detect all possible single point mutations leading to a missense substitution at that codon. Following initial validation we applied this assay to 124 cases of B cell lymphoma with a germinal center phenotype.

SNaPshot genotyping was performed in 96-well plates and is based on a one-tube protocol validated for clinical use in the Massachusetts General Hospital Diagnostic Molecular Pathology Laboratory. Genomic DNA containing *EZH2* codon 641 was PCR amplified from TNA using the forward primer 5′-AGTTAGTATATACAATGCCACCTG-3′ and reverse primer 5′- CTCTAGCATCTATTGCTGGC-3′, followed by a cleanup reaction with shrimp acid phosphatase and exonuclease 1 (USB). The SNaPshot single-nucleotide extension reaction was performed using SNaPshot Ready Reaction mix (Applied Biosystems, Foster City, CA) and 4 multiplexed extension primers designed to interrogate the first two nucleotides of *EZH2* codon 641 on both the coding and noncoding strands. Following a final shrimp acid phosphatase cleanup reaction, extension products were evaluated by capillary electrophoresis, using an Applied Biosystems 3500×L Genetic Analyzer. Extension primers used for initial screening of all samples were as follows:

EZH2_c.1921F.v1: 5′-ACTGACTGCAGAAAAATGAATTCATCTCAGAA-3′

EZH2_c.1922F.v1: 5′-GACTGACTGACTGACTGACTGACTGACTGCAGAAAAATGAATTCATCTCAGAAT-3′

EZH2_c.1921R.v1: 5′-GACTGACTGACTGACTGACTGACTGTGCCTTACCTCTCCACAGT-3′

EZH2c.1922R.v1: 5′-AGTGCCTTACCTCTCCACAG-3′

A final set of optimized extension primers was used to confirm all detected mutations:

EZH2_c.1921F.v2: 5′-CAGAAAAATGAATTCATCTCAGAA-3′

EZH2_c.1922F.v2: 5′-AGTCAGTCAGTCAGAAAAATGAATTCATCTCAGAAT-3′

EZH2_c.1921R.v2: 5′-AGTCAGTCAGTCAGTCAGTCAGTCAGGTGCCTTACCTCTCCACAGT-3′

EZH2_c.1922R.v2: 5′-AGTCAGTCAGTCAGTCAGTCAGTCAGTCAGTCAGTCAGTGCCTTACCTCTCCACAG-3′

All transcript sequence references are to the *EZH2* coding sequence of RefSeq isoform C, NM_001203247.1, as explained subsequently.

### Fluorescence in-situ hybridization

All cases of unknown *BCL2* status were assessed for the presence of *BCL2* rearrangement on 2–5 micron FFPE sections using the Vysis LSI *BCL2* Dual Color Break Apart Rearrangement Probe (Abbott Molecular, Des Plaines, IL). Cases were scored as *BCL2*-rearranged (BCL2-R) on the basis of detecting unambiguous probe separation or splitting in at least 15% of observed cells (50-cell count) as scored by two independent observers (M.N. and R.J.H.R.).

### Statistical analysis

Statistical significance of differences in EZH2 mutation frequency between lymphoma groups was calculated by the two-tailed Fisher's exact test using GraphPad QuickCalcs (GraphPad Software Inc., La Jolla, CA). Statistical significance was defined as p<0.05.

### Cloning of EZH2 coding sequence

The *EZH2* coding sequence was PCR amplified from pooled human bone marrow cDNA (Clontech) using the primers 5′-GGGGACAAGTTTGTACAAAAAAGCAGGCTGCACCATGGACTACAAGGACGACGATGACAAAGGCCAGACTGGGAAG-3′ and 5′-GGGGACCACTTTGTACAAGAAAGCTGGGTTAAGGCAGCTGTTTCAGAGG-3′ which encode an N-terminal FLAG tag. PCR products were cloned into pDONR221 and confirmed by sequencing. A clone matching the coding sequence of *EZH2* RefSeq isoform C was subcloned into pCDNA3.2/V5-DEST using the Gateway recombination system (Invitrogen). Codon 641 and 689 point mutations were generated in pENTRY vectors using the QuickChange Lightning kit (Stratagene) and were subcloned into pCDNA3.2/V5-DEST. SET domain-truncated *EZH2* (EZH2ΔSET) was generated by PCR subcloning with the C-terminal primer 5′-GGGGACCACTTTGTACAAGAAAGCTGGGTTCATGCCAGCAATAGATGCTTTTT-3′ as previously described. [7]

### EZH2 transgene expression and western blotting

*EZH2* vectors were transfected into sub-confluent NIH-3T3 murine fibroblasts with Fugene HD reagent (Roche) according to the manufacturer's instructions. Cells were harvested 72 hours later and conventional western blotting was performed on whole-cell extracts (targets: EZH2, Beta-actin), or acid-extracted histones (targets: total H3, H3K27me3, H3K27me2, H3K27me1, H3K9me3). Ponceau S was used to confirm transfer of equivalent total protein levels in all lanes prior to antibody binding. The antibodies used for western blot are as follows:

Beta-actin – Abcam (Cambridge, MA), Cat# ab8827 (rabbit)

EZH2 – Active Motif (Carlsbad, CA), Cat# 39639 (rabbit)

H3 (total) – Abcam, Cat# ab1791 (rabbit)

H3K9me3 – Abcam, Cat# ab8898 (rabbit)

H3K27me1 – Active Motif, Cat# 39377 (rabbit)

H3K27me2 – Abcam, Cat# ab24684 (rabbit)

H3K27me3 – Millipore (Billerica, MA), Cat# 07-449 (rabbit)

### RNA-seq analysis

Published RNA-seq data for DLBCL cell lines Oci-Ly1, Oci-Ly7, and Oci-Ly19 (SRA accession SRP001599) [14] were aligned to the human genome (Hg19) using TopHat [15] and analyzed with the Integrative Genomics Viewer. [16]

## Results

**Figure 1:**
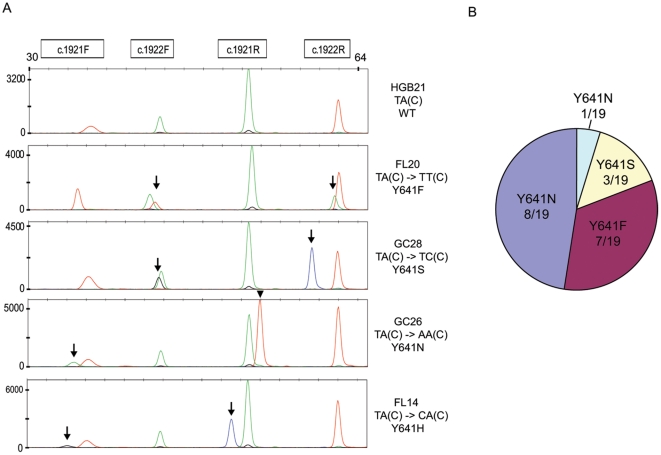
SNaPshot detection of *EZH2* codon 641 mutations. A: Representative electropherograms for SNaPshot genotyping of *EZH2* in B cell lymphomas. Five representative tracings are shown, representing lymphomas with wild-type *EZH2* codon 641 and each of the four distinct missense substitutions detected in this study. Two complementary mutant peaks (arrows) appear in each mutant sample, corresponding to detection of the missense substitution by both the forward and reverse primers at the same nucleotide position. B: Distribution of *EZH2* codon 641 missense mutations detected by SNaPshot.

The results of SNaPshot genotyping in 124 germinal center B cell lymphomas are shown in Table 1. Overall, we detected *EZH2* codon 641 mutations in 19/124 (15%) cases. All mutations were genotyped redundantly by the corresponding forward and reverse extension primers, and were detected in independent screening and confirmatory runs. Detected mutations included all of the four most common reported variants (Figure 1). No novel mutations were identified.

**Table 1:** *EZH2* and *BCL2* aberrations in germinal center lymphomas by category.

Classification	*EZH2* mutated	*BCL2* rearranged
**All follicular lymphoma (n = 55)**	**12 (22%)**	**43 (78%)**
Grade 1–2, low PI (n = 23)	7 (30%)	21 (91%)
Grade 1–2, high PI (n = 18)	4 (22%)	15 (83%)
Grade 3 (n = 14)*	1 (7%)	7 (50%)
**All GCB-DLBCL (n = 35)**	**5 (14%)**	**12 (34%)**
Pure DLBCL, no prior lymphoma (n = 23)**	1 (4%)	6 (26%)
Concurrent DLBCL and grade 3 FL (n = 9)	4 (44%)	4 (44%)
Prior low-grade FL (n = 1)	0 (0%)	1 (100%)
Prior low-grade lymphoma, not FL (n = 2)	0 (0%)	1 (50%)
**“Double hit” B cell lymphomas (n = 11)** ***	**2 (18%)**	**11 (100%)**
De novo (n = 7)	1 (14%)	7 (100%)
History of follicular lymphoma (n = 4)	1 (25%)	4 (100%)
**Burkitt lymphoma (n = 23)**	**0 (0%)**	**0 (0%)**

*Includes grades 3A (n = 11) and 3B (n = 3). All grade 3B cases lacked both EZH2 mutation and BCL2 rearrangement.

**A single case within this group lacking both an *EZH2* mutation and a *BCL2* rearrangement had concurrent follicular lymphoma *in situ*.

***All “double hit” B cell lymphomas had rearrangements of the *MYC* and *BCL2* loci.

The frequency of *EZH2* mutations within the various lymphoma subtypes is given in Table 1. Among the cases screened, *EZH2* mutations were detected with relatively high frequency in FL (12/55, 22%). There was no difference in *EZH2* mutation frequency between the low-PI and high-PI subgroups of grade 1–2 FL (low PI 7/23, 30%; high PI 4/18, 22%, p = 0.73) or between *EZH2* mutation status and morphologic FL grade (Grade 1–2 11/41, 27%; Grade 3 1/14, 7%, p = 0.16). Within the grade 3 FL group, 3 cases were graded as 3B, and none of these cases showed an *EZH2* mutation. We also detected *EZH2* mutations in the GCB-DLBCL (5/35, 14%) and DHL (2/11, 18%) groups, but not in any case of BL (0/23). Four of the five DLBCL *EZH2* mutations belonged to a group of nine DLBCL cases that had concurrent diagnoses of grade 3 FL.

**Figure 2:**
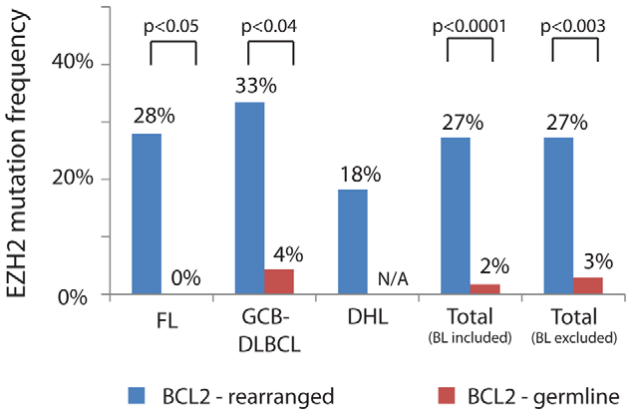
Frequency of *EZH2* mutations in germinal center lymphomas by *BCL2* status. Within each class of germinal center lymphoma, *EZH2* mutations were significantly more frequent in cases with rearrangements of *BCL2*. Burkitt lymphoma (not shown) universally lacked both aberrations. The two total columns at right include all lymphomas in the study (fourth column) or all lymphomas except BL (fifth column) – see table 1 for subtype distribution. Abbreviations: FL = follicular lymphoma, GCB-DLBCL = germinal center B-cell phenotype diffuse large B-cell lymphoma, DHL = “double hit” high-grade B-cell lymphoma (*MYC* and *BCL2* rearranged), BL = Burkitt lymphoma.

We used FISH to screen for *BCL2* rearrangements in all of our lymphoma cases that were not previously tested for this aberration. As expected, *BCL2* rearrangements were detected in the majority of FL (78%) and in a smaller proportion of GCB-DLBCL (34%) (Table 1). We found these mutations to occur more frequently in *BCL2*-rearranged lymphomas than in lymphomas lacking a *BCL2* rearrangement. The increased frequency of *EZH2* mutations was statistically significant in the FL group (28% of BCL2-R cases versus 0% of BCL2-WT cases, p<0.05), the DLBCL group (33% of BCL-R cases versus 4% of BCL2-WT cases, p<0.04), and across all lymphoma types excluding BL, which universally lacked both *BCL2* rearrangements and *EZH2* mutations (27% of BCL2-R cases versus 3% of BCL2-WT cases, p<0.003) (Figure 2).

To determine the function of lymphoma-associated *EZH2* mutations in a cellular context, we PCR cloned the *EZH2* coding sequence from human bone marrow cDNA. Of the 7 independent clones we sequenced, all contained the exon 8–9 splice junction seen in *EZH2* Refseq isoform C (NM_001203247), corresponding to the Y641 codon designation used in the initial description of the lymphoma mutation, [4] while no clones contained the exon 8–9 splice junction seen in the slightly longer *EZH2* Refseq isoform A (NM_004456), which has been used as a reference transcript in some later work, resulting in designation of the same residue as Y646. [17], [14] Alignment of published RNA-seq data for GCB-DLBCL cell lines Oci-Ly1, Oci-Ly7, and Oci-Ly19 demonstrated that the vast majority of reads spanning the *EZH2* exon 8–9 splice junction support the splice junction found in isoform C, and not the junction found in isoform A (Figure S1 and data not shown). Of the available spliced expressed sequence tags (ESTs) in GenBank (release 185.0, accessed August 30, 2011) derived from various tissues, 39/41 sequences match the exon 8–9 splice junction found in isoform C.

**Figure 3:**
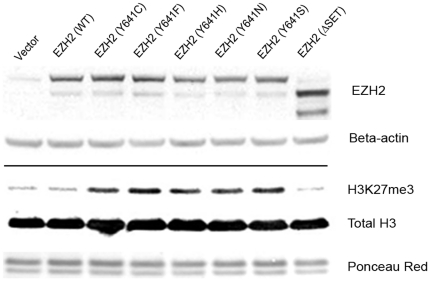
Western blot of mutant EZH2 expression in NIH-3T3 fibroblasts. Transgenic expression of vectors encoding lymphoma-associated *EZH2* codon 641 mutants, but not wild-type or SET domain-truncated (ΔSET) *EZH2*, leads to a consistent increase in global trimethylation of H3K27 compared to vector control. Western blot antibodies are listed on the right. Ponceau S depicts total protein at ∼15 kDa on acid-extract blots.

We generated expression vectors encoding wild-type *EZH2*, each of the five reported lymphoma-associated codon 641 mutants, and two well-characterized synthetic methyltransferase loss-of-function mutants, the point mutant H689A, [18] and a truncation mutant lacking the C-terminal methyltransferase (SET) domain. Western blotting of acid-extracted histones from transiently transfected NIH-3T3 fibroblasts showed no difference in global H3K27me3 levels between cells transfected with empty vector, wild-type *EZH2*, *EZH2ΔSET* (Figure 3), or *EZH2 H689A* (data not shown). In contrast, there was a marked increase in global H3K27me3 levels in cells transfected with each of the five lymphoma-associated mutants. There was no measurable difference in global levels of H3K27me2, H3K27me1, and H3K9me3 between the transfectants (Figure S2).

## Discussion

Our findings confirm and extend prior work regarding the prevalence of *EZH2* mutations in B cell lymphomas and the molecular function of these mutations. Clinical detection of oncogenic point mutations conferring enzymatic gain-of-function has been a fruitful strategy for triaging patients to molecularly targeted therapy in several cancer types, including lung adenocarcinoma (*EGFR* mutations) [19] and melanoma (*BRAF* V600E mutations). [20] While specific small-molecule inhibitors of EZH2 are not yet clinically available, there is ongoing interest in the development of such agents. [5] Our SNaPshot-based assay reproducibly detected *EZH2* mutations in a substantial proportion of germinal center lymphomas from routine FFPE clinical specimens. The absence of *EZH2* mutations in BL highlights both the biological specificity of the *EZH2* codon 641 mutation for certain lymphoma subtypes, and the technical specificity of our assay.

In comparison to prior studies, we detected a relatively high rate of *EZH2* mutations in FL (22% overall and 27% in the common grade 1–2 subtype) compared to prior reports (7% [4] and 12% [21] overall). This may be due, in part, to the higher sensitivity of SNaPshot compared to Sanger (chain terminator) sequencing for detection of mutations in heterogeneous tumor tissue. [13] Our results are consistent with those of a recent study which used deep-sequencing techniques (genome, exome, and RNA sequencing) on a small number of follicular lymphomas and reported *EZH2* mutations in 4/13 cases (31%). [14] Thus, the true rate of *EZH2* mutations in follicular lymphoma may be higher than originally reported. *EZH2* mutations were not enriched in more proliferative subgroups of FL (low grade/high PI FL and grade 3 FL), suggesting that *EZH2* mutations are not associated with known aggressive subtypes of FL. Larger studies with clinical follow-up will be required to determine whether this mutation carries independent prognostic significance in FL.

In contrast to the findings in FL, we detected mutations in a relatively low proportion of GCB-DLBCL (14%) compared to the original report (22%) [4]. Our use of an immunophenotypic surrogate for the GCB-DLBCL gene expression profile could partly explain this discrepancy, although our findings are similar to those of another recent study, which found *EZH2* mutations in 14% of a cohort of 63 microarray-defined GCB-DLBCL. [22] Interestingly four of the five *EZH2* mutations detected in the GCB-DLBCL cohort occurred in a subgroup of nine cases in which the diagnostic biopsy showed a large cell lymphoma with areas of both follicular and diffuse growth, resulting in concurrent diagnoses of DLBCL and grade 3 FL by current WHO criteria. [1] None of these nine patients had a prior history of follicular lymphoma, and these cases appear to represent *de novo* high-grade germinal center phenotype lymphomas with partial residual folliculotropism. This finding raises the possibility that *EZH2*-mutated DLBCL may have characteristic morphological and biological features, and merits further investigation.

The *EZH2* codon 641 mutation was first identified through whole-genome sequencing of a follicular lymphoma that lacked a t(14;18) *IGH*-*BCL2* rearrangement. [4] However, we found frequent, though not universal, co-occurrence of *EZH2* codon 641 mutations with rearrangements of *BCL2*. This implies that *EZH2* mutations do not substitute for *BCL2* rearrangement, but rather represent a functionally distinct oncogenic “hit” in germinal center B cell lymphomagenesis. Targeted inhibitors of BCL2 have been developed, with some advancing into clinical trials. [23] Our finding that many lymphomas show oncogenic activation of both BCL2 and EZH2 raises the possibility that combined BCL2 and EZH2 inhibition may represent a potential therapeutic strategy for many patients.

BL is a prototypical germinal center-derived lymphoma, but shows substantial biologic, pathogenic, and epidemiologic differences from FL and DLBCL. [1] Our findings suggest that *EZH2* mutations are rare or absent in this lymphoma type. We did detect *EZH2* mutations in *MYC* and *BCL2*-rearranged “double hit” high grade B cell lymphomas, which often show morphologic, genetic, and immunophenotypic features intermediate between DLBCL and BL, and are associated with a poor prognosis [12]. Large-scale integrative studies including gene expression profiling have suggested that the relative paucity of large-scale genomic imbalances (“genetic complexity”) can be helpful in distinguishing “true” BL from other high-grade B cell lymphomas. [24] Our results suggest that *EZH2* mutation status could also potentially contribute to this distinction.

The results of our mutant EZH2 expression experiments indicate that the mutant protein conferred a gain-of-function phenotype as evidenced by increased generation of the H3K27me3 histone mark, the mechanism of which has been demonstrated by others. [5] Interestingly, the mutation that was most common in our series, Y641N, was estimated to be the most potent of the four common mutations at generating H3K27me3 in the heterozygous state, based on *in-vivo* enzyme kinetics, [5] while Y641H, the least common mutation in our series, was predicted to be least potent. These results suggest that more potent mutations may undergo stronger oncogenic selection. Our novel demonstration of a similar gain-of-function activity for the Y641C mutant in an overexpression assay is consistent with the reported occurrence of this mutation in B cell lymphoma, [4] in which most *EZH2* mutations are thought to confer gain-of-function. However, the Y641C mutation has also been reported in an acute myeloid leukemia cell line, despite the fact that many reported *EZH2* mutations in myeloid tumors are clearly inactivating. [17] These findings suggest the possibility of a more complex function for some myeloid *EZH2* mutations, and highlight the need for further functional investigation of these mutant proteins. While the H3K27me3 modification is known to have a repressive effect on gene expression, the precise genomic targets that are affected by the mutant protein during lymphomagenesis and confer selective advantage have not been identified.

For our transgenic work, we used a cDNA clone for *EZH2* with a coding sequence matching that of the original reported *EZH2* coding sequence [25] and *EZH2* Refseq isoform C (NM_001203247). Our results suggest that this is the predominant long splice form of *EZH2* in lymphoma and other tissues, and for this reason we prefer to designate the *EZH2* codon affected by recurrent mutations in B cell lymphoma as Y641, rather than Y646. Shorter *EZH2* transcripts produced by variant splicing of exons 3 and 4 are also well supported by ESTs and other public data, and are of uncertain significance.

In conclusion, we have developed a clinically applicable assay for sensitive detection of *EZH2* codon 641 mutations in FFPE tissue, and demonstrate frequent occurrence of these gain-of-function mutations in the full biological spectrum of *BCL2*-rearranged germinal center phenotype B cell lymphomas, but not in Burkitt lymphoma.

## Supporting Information

Figure S1 RNA-seq analysis of *EZH2* transcript splice variants in the DLBCL cell line Oci-Ly1. Alignment of RNA-seq data from the DLBCL cell line Oci-Ly-1 [14] to the 3′ splice junction of *EZH2* exon 8 shows that the vast majority of transcripts contain the splice junction seen in *EZH2* isoform C (NM-001203247), as marked by the black arrow. Only rare reads, marked with a red arrow, support the alternate splice site seen in *EZH2* isoform A (NM_004456), which encodes an additional 5 amino acids. Findings in the DLBCL cell lines Oci-Ly7 and Oci-Ly19 (not shown) were similar.
(TIF)

Figure S2 Western blot of EZH2 mutant overexpression in NIH-3T3 fibroblasts. Transgenic overexpression of lymphoma-associated *EZH2* codon 641 mutants was not associated with differences in the global levels of histone marks H3K27me1, H3K27me2, and H3K9me3 compared to vector control, wild-type, or SET domain-inactivated *EZH2*.
(TIF)
