# Supplementary figures and images for: EZH2 Codon 641 Mutations are Common in BCL2-Rearranged Germinal Center B Cell Lymphomas
Source: PLoS One. 2011 Dec 14;6(12):e28585. doi: 10.1371/journal.pone.0028585 (PMC3237460; doi:10.1371/journal.pone.0028585)

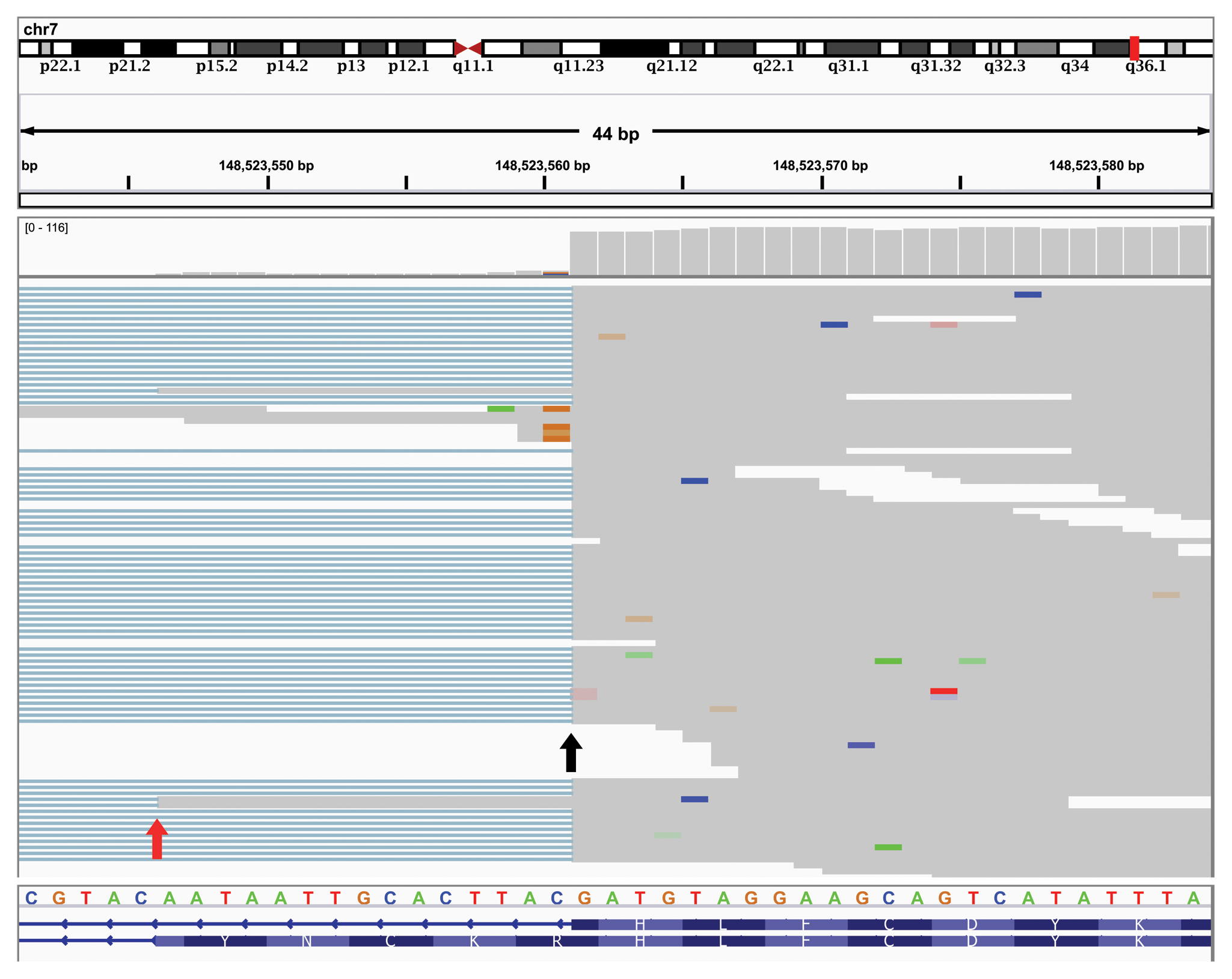

Supplement: Figure S1 — RNA-seq analysis of EZH2 transcript splice variants in the DLBCL cell line Oci-Ly1. Alignment of RNA-seq data from the DLBCL cell line Oci-Ly-1 [14] to the 3′ splice junction of EZH2 exon 8 shows that the vast majority of transcripts contain the splice junction seen in EZH2 isoform C (NM-001203247), as marked by the black arrow. Only rare reads, marked with a red arrow, support the alternate splice site seen in EZH2 isoform A (NM_004456), which encodes an additional 5 amino acids. Findings in the DLBCL cell lines Oci-Ly7 and Oci-Ly19 (not shown) were similar. (TIF) [file pone.0028585.s001.tif]

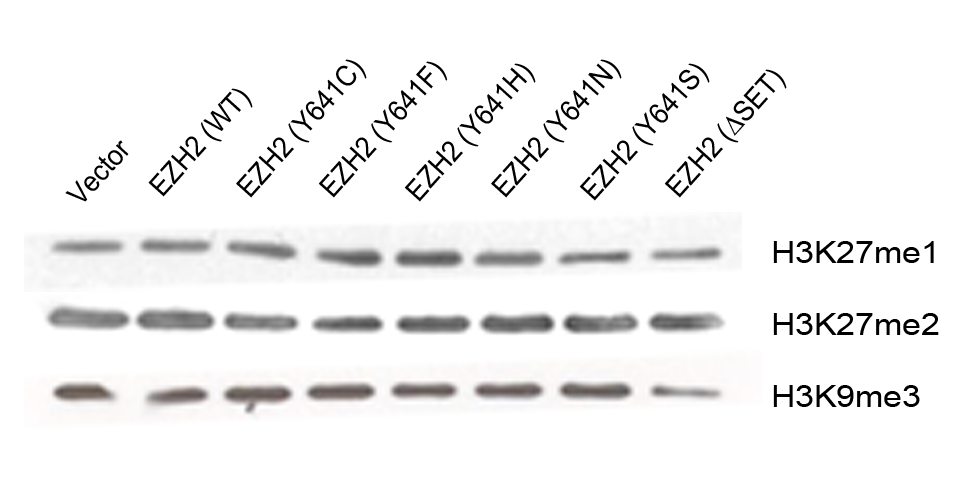

Supplement: Figure S2 — Western blot of EZH2 mutant overexpression in NIH-3T3 fibroblasts. Transgenic overexpression of lymphoma-associated EZH2 codon 641 mutants was not associated with differences in the global levels of histone marks H3K27me1, H3K27me2, and H3K9me3 compared to vector control, wild-type, or SET domain-inactivated EZH2. (TIF) [file pone.0028585.s002.tif]
